# Letter from Samuel Cole, M. D., Berlin, Prussia

**Published:** 1868-08-15

**Authors:** Sam’l Cole

**Affiliations:** Berlin


### *FOREIGN CORRESPONDENCE.*

Berlin, *June 29th*, 1868.

*To the Editor of the Chicago Medical Journal:*

Dear Sir,—Since my last from Vienna, I have continued on my tour through Prague and Leipsic to Berlin, and can not refrain from mentioning to you a few of the many objects of scientific worth I was fortunate enough to meet with. Among many men of reputation, I had the pleasure of an introduction to Prof. Henry, of Vienna, and Prof. Ludwig, of Leipsic. Both gentlemen are physiologists of rank, and have constructed apparatuses for injection; rendering the delicate operation of filling fine capillaries easy, and practicable for all. As long as this was performed by the hand and syringe, it was very difficult to obtain good specimens; for, unless particularly skillful, (as, for instance, Prof. Thiersch, of Leipsic), the pressure was not constant, now stronger, now weaker, when too strong, causing extravasation; when too weak, plugging the beginning of the capillary. The two physiologists above mentioned, experiencing the necessity of something more reliable than the untutored hand, constructed apparatuses by means of which fluids are propelled through the vessels by the constant pressure of a column of mercury of a determined height. The credit of the discovery belongs to to Ludwig; that of the best improvement to Hering. The apparatus of the former has this disadvantage—that, at the time of filling in the injecting mass, the mercury must be evacuated, and thus a considerable quantity of this expensive fluid is lost. Prof. Hering has arranged, by a number of globes and tubes, to make repeated use of the same column of mercury, not a particle being wasted. The apparatus, though apparently complicated, is very simple and ingenious. Both gentlemen, with the characteristic kindness of *German Professors*, allowed us to make use of their apparatus under their guidance.

There is an animated controversy amongst German oculists in regard to the superiority of the extracting of cataracts by section through the *cornea* or the *sclerotic*. Prof. Hasner, of Prague is the strongest advocate for the corneal wound; Prof. Knapp one of the vindicators of the scleral. As, in the course of our trip, we stopped at Prague, the two gentlemen met and concluded to operate according to their several opinions in order to discuss the subject, and, if possible, throw some light upon it. Prof. Knapp performed the operation with his accustomed skill, the cataract-knife of Von Graefe being used; the lens was delivered with ease by simultaneous pressure on the posterior lip of the wound with one spoon, and on the lower part of the corner with another. Prof. Hasner, who, until then had denied the possibility of making a linear wound, acknowledged this one to be perfectly so, there being no height of flap. The Professor had operated on a case previously by the usual flap operation, and, according to his method, performed the puncture of the hyaloïdea after the escape of the lens. Both patients could distinguish objects after the operations, so that by this operative demonstration nothing was gained, but the possibility of exact execution of both methods; which, of the two, merits our preference, can only be deduced from ultimate statistical results. Most German oculists have abandoned the flap operation for the new method, and all who have tried the latter on a larger scale, so as to acquire skill in performing it, are enthusiastic in its praise, asserting that the results are decidedly better than those of any other method. The puncture of the hyaloid membrane, as performed by Prof. Hasner, causes a small portion of the vitreous to flow into the anterior chamber, and constitutes his modification of the flap operation. According to his views, it has the advantage of removing remaining portions of the lens and capsule from the centre of the pupil. He claims to obtain a much clearer pupillary field than if remnants of the lens and capsule were allowed to remain. In the operation above mentioned, the vitreous filled the anterior chamber after the puncture had been made, the iris thus becoming removed from the cornea, and the pupillary field becoming clear. No vitreous escaped from the corneal wound. Prof. Hasner then showed us a number of cases which had been operated according to his method. In two, the pupils were as clear as if the capsule had been extracted with the lens. On further examination, we found some patients with coloboma of the iris, consequent to prolapsus iridis after the operation; in others, pupillary membranes, more or less dense (pseudoplastic membranes from iritis) were present; and, in one case, suppurative iritis threatened to destroy the eye entirely. These accidents can not be ascribed to the puncture of the hyaloïdea, since they are consequences of the flap operation, not at all rare when the vitreous is left untouched. On the other hand, such perfect clearness of the pupil as we saw in the two cases first mentioned, is only exceptionally met with when the puncture is not made. What would be the result of a combination of Hasner's method of puncture of the hyaloïdea with the modified linear operation, as performed by A. von Graefe ?

And now, before leaving the subject of Opthalmology, I must not forget Stellwag von Carion. He showed us a number of specimens, the most remarkable of which was a fungoid growth in the conjunctiva of a boy. After having been cauterized by another physician, this growth, primarily orbital, soon perforated the globe, and became intraocular. Prof. Stellwag recovered a portion of the orbital tumor. The remainder shrunk, and at the expiration of three years, there had been no recidive. Under the microscope the growth proved to be a granulation tumor. It shows how luxuriant granulations may stimulate a character of malignancy by undue irritative treatment.

Stellwag lays particular stress on the fact, that in the great majority of cases, convergent strabismus is due to hypermetropia, and says that the operation is ineffectual very often when convex glasses are not applied afterward, asserting that the squint will disappear, if this error of refraction be corrected by suitable convex glasses. The resort to these glasses he prefers deferring to the fifth year, and considers it inadequate after puberty. In the latter case, he first operates and then makes use of glasses, to insure the permanency of the result. The ophthalmologists of Germany prefer the latter method in all cases, both before and after puberty, and although he is supported in his views (Donders), it still remains a problem whether they will become generally accepted or not.

In his wards we were also fortunate enough to see a number of cases of soft cataract, in which he had performed a modified flap operation instead of the usual discission. It consists in making a short flap and small wound about one line anterior to the margin of the cornea, and thus extracting the nucleus of the opaque lens, whilst the soft, cortical substance is permitted to peel off. The greater portion can be evacuated afterward, and the remainder is dissolved in the aqueous humor. He has not operated a sufficient number of cases to assert that it is preferable to the methods in use, but claims to have obtained favorable results. This procedure is no new one. It is the method of the so-called linear extraction, performed in England some years ago by Gibbon, Critchett and others; afterward in Germany by Von Graefe, and a number of German opthalmologists. The only difference is in the instrument employed—Stellwag making his incision with an ordinary cataract-knife, instead of the lance-knife. The indications, every manipulation during the operation, and the after-treatment, are identical. The operation, however, is not commendable, as the danger of anterior synechiæ, and its consequences (Glaucoma), is certainly increased, and, in many instances, must inevitably follow. This method has been almost completely abandoned in favor of discission, or extraction through the scleral border.

In Vienna, we took a high interest in the clinical and pathological anatomical demonstrations which Prof. Politzer, (one of the most renowned authorities in aural surgery), was kind enough to make during a whole week. His ingenious method of driving air into the tympanic cavity through the eustachian tube during the act of deglutition, I need only mention here, as it is well known throughout the medical world. On a large number of patients we were witnesses of its practical utility in furthering diagnosis, and as a therapeutic agent. Another excellent method of investigation, introduced by him, consists in determining the cause of deafness by means of a tuning-fork placed upon the middle of the cranium. Some patients, when subjected to this proceeding, affirm that they distinguish the sound better with the deaf ear; others, on the contrary, with the unaffected ear. According to Prof. Politzer, this is due to obstructions in the tympanic cavity and the external meatus, and to thickening of the tympanum itself; in this manner, the expansion of the undulations of sound to the air being more or less interrupted. When the tuning-fork is placed on the middle line of the cranium, the undulations are conducted through the cranial bones to the labyrinth, and by the fenestra rotunda, and, perhaps, ovalis, to the tympanic cavity, the tympanum, external meatus, and air. If the tympanum be thickened, or if there be an obstacle in the cavity of the tympanum, or meatus auditorius externus, the undulations of sounds are transmitted to the air only in part, whilst in part they are reflected on the fenestra and bony walls of the labyrinth. This is confirmed by an artificial ear constructed by the Professor. The tympanic cavity, the ossicles, tympanum, external meatus, and the beginning of the labyrinth, are represented in it, and he varies the effect by means of a diaphragm (for the pathological thickening of the external parts of the ear), which he introduces into the meatus auditorius externus. I dwelt rather long on this subject because it is new, and highly important in the diagnosis of the cause of deafness, whether it lies in the labyrinth itself or external to it.

Prof. Politzer, among many other interesting pathological specimens, also kindly demonstrated diseases of the tympanum, different kinds of inflammatory products within the tympanic cavity, caries and purulent destruction of the bony parts of the organ of hearing, and their sad consequences; suppurative meningitis and thrombus of the cranial sinuses.

It would lead me too far to describe these specimens in detail, and I should owe your readers an apology for the minuteness with which I have entered into the above description, were it not that the interest and novelty of this method of diagnosticating diseases of the ear bears the excuse in itself.

Yours, truly,

SAM'L COLE, M.D.

